# Comparison of Hero 642 and K3 rotary nickel-titanium files in curved canals of molars and a systematic review of the literature

**DOI:** 10.3892/etm.2014.1853

**Published:** 2014-07-18

**Authors:** HUA-XIONG CAI, HUI-LING CHENG, JIE-WEN SONG, SU-YA CHEN

**Affiliations:** 1 Department of Operative Dentistry and Endodontics, Guanghua School and Hospital of Stomatology and Guangdong Provincial Key Laboratory of Stomatology, Sun Yat-sen University, Guangzhou, Guangdong 510055, P.R. China; 2 Department of Stomatology, The Second Affiliated Hospital, Sun Yat-sen University, Guangzhou, Guangdong 510120, P.R. China; 3 Department of Stomatology, Eighth People’s Hospital of Guangzhou, Guangzhou, Guangdong 510060, P.R. China

**Keywords:** canal curvature, canal strengthening, Hero 642 files, K3 files, root canal preparation

## Abstract

The aim of the present study was to compare the root canal preparation ability of rotary nickel-titanium (NiTi) Hero 642 and K3 files in curved mandibular or maxillary molars. A total of 40 extracted mandibular molars with two separate mesial canals, an apical width of approximately size ≤15 and a root canal curvature of 15–30° were randomly divided into two groups and instrumented using Hero 642 (n=20) or K3 files (n=20). Canal straightening, working length, transportation, cross-sectional area, minimum dentin thickness and the canal angle curvature degree were examined, and a systematic review of the literature was conducted. No statistically significant differences were observed between the two groups with regard to the mean degree of straightening, mean change in working length, mean transportation, amount of dentin removed or remaining minimum dentin thickness (P>0.05). The canal angle curvature decreased in the two groups postoperatively. The systematic review identified six studies, and overall the two files performed similarly in the majority of categories examined. Therefore, the rotary NiTi Hero 642 and K3 files demonstrated comparable shaping abilities and maintenance of working length.

## Introduction

Rotary nickel-titanium (NiTi) instruments are favored over stainless steel hand files for root canal treatment due to the superior quality of canal preparation (1). Although NiTi systems have similar features, they differ in their cross-sectional and shank design. The design of the instruments, including the cutting angle, number of blades, tip design, conicity and cross-section, directly influences the flexibility, cutting efficacy and torsional resistance of the instrument (2), as well as their performance in narrow or wider canals (3,4).

Hero 642 rotary instruments (Micro-Mega, Besençon, France) are comprised of a NiTi alloy, and incorporate instruments with .06, .04 and .02 tapers (T) in sizes 20, 25 and 30, with additional .02 T in sizes 35 and 40. The graduated tapers initially prepare the coronal portion of the root canal; the final shape being created by merging the apical and coronal preparations (5). The Hero 642 file contacts the root canal wall dentin via blade contact, and has a triple helix cross-sectional design with three cutting edges with a positive rake cutting angle that provides a high cutting efficiency and is less prone to torsional fatigue.

The K3 file (SybronEndo, West Collins, CA, USA) contacts the root canal wall dentin via radial plane contact, and has a large cross-sectional area with a negative rake angle as the cutting angle, a flattened non-cutting tip and an asymmetrical constant tapered active file design with a variable helical flute and a variable core diameter (6). The proportion of the core diameter to the outside diameter is greatest at the tip and decreases uniformly towards the shank, resulting in greater flute depth and increased flexibility. The K3 instruments have non-cutting tips which minimize the risks of ledging, zipping, perforations and canal transportation, however, are more prone to torsional fatigue (6).

Numerous studies have compared NiTi instruments in extracted molars; however, the investigations are primarily limited to extracted teeth from Caucasian populations (7–11). The aim of the present study was to examine root canal preparations of Hero 642 and K3 rotary instruments in molars from southern Chinese individuals, and to perform a systematic review to summarize the observations of previous studies comparing Hero 642 and K3 files in order to obtain a more comprehensive understanding of the differences between the techniques.

## Materials and methods

### Specimen selection and preparation

Extracted first and second mandibular molars with intact crowns and fully-formed apices were collected from the Department of Stomatology of the Second Affiliated Hospital of Sun Yat-Sen University (Guangzhou, China). Soft tissues and hard deposits on the surface of the teeth were removed by scaling; the teeth were placed in 5% sodium hypochlorite solution for 1 h to facilitate cleaning. The teeth were then stored in normal physiological saline until required. A total of 40 mandibular molars were selected based on the following criteria: Two separate mesial canals, apical width of approximately size ≤15 (evaluated using files until size 15) and root canal curvature of 15–30° (determined by radiographs). The teeth were randomly assigned to two groups of 20 teeth each (groups A and B) using a randomization table.

**Figure 1:**
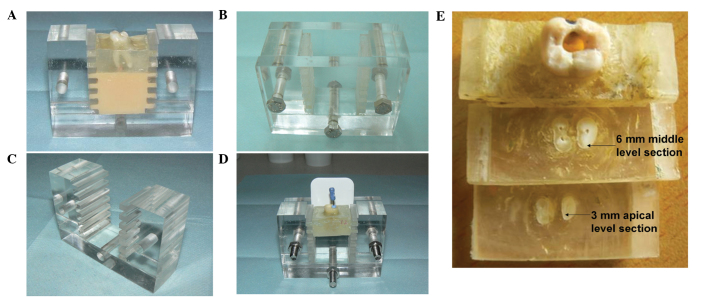
Representative images showing (A) a tooth embedded in clear epoxy resin; (B) the muffle block system; (C) the middle section of the muffle system revealing the grooves; and (D) the file in the canal. Radiographs were obtained in buccolingual and mesiodistal directions. X-ray films were placed behind the teeth for radiography. (E) Two observation levels were selected, one was 3 mm from the apex, defined as the ‘apical level’ and the other was 6 mm from the apex, defined as the ‘middle level’.

Conventional access cavities were formed, and the mesial canals were controlled for patency using a size 10 K-file (Dentsply Maillefer, Ballaigues, Switzerland). Grooves were established in the walls of the middle section to allow the removal and exact repositioning of the complete tooth blocks or sectioned parts of the tooth. All the teeth were shortened to a length of 17±2 mm by trimming the occlusal surfaces. The root tips were sealed with a thin film of wax and access cavities with cotton pellets, and the teeth were embedded in clear epoxy resin (Fig. 1A). A modified Bramante muffle system (12,13) was used to embed the teeth for root canal preparation (Fig. 1B and C). Following polymerization of the resin, the wax was removed and the apical foramen was exposed. To determine the working length, a size 10 K-file was introduced into the canal until the tip was just visible at the apical foramen. The working length was calculated to be 1 mm short of this distance.

To determine the angle of curvature, standardized radiographs were captured prior to instrumentation with a size 10 K-file in the canal (Fig. 1D), and following instrumentation with a size 30 K-file in the canal. All X-ray imaging was performed by the same experienced radiographer using a consistent orientation of the specimens to ensure reproducibility. The radiographs were obtained in the buccolingual and the mesiodistal directions. The films were then digitized using a scanner (Canoscan Lide 25; Canon, Inc., Tokyo, Japan) and input into software (AutoVue SM Pro; Cimmetry Systems, Inc., Cambridge, MA, USA) for measurements.

**Figure 2:**
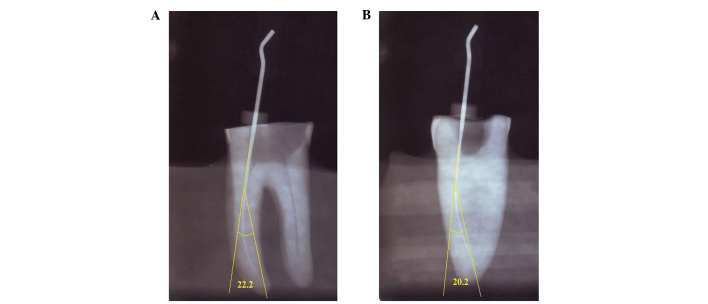
Measurement of the angle of curvature from the (A) mesiodistal and (B) buccolingual radiographic views.

The clinical and proximal angles of curvature were measured according to Schneider’s method (14) (Fig. 2), and the true angle of curvature (TAC) was calculated using Samyn’s formula (15) as follows:

$$\text{TAC}=\text{Arc Tan }{\left[{\left(\text{TanC}\right)}^{2}+{\left(\text{TanP}\right)}^{2}\right]}^{1/2}$$

where C was the clinical angle and P was the proximal angle. The angle of curvature was calculated in the direction of greater curvature.

### Canal instrumentation

Group A were assigned for preparation with Hero 642 instruments (Micro-Mega, Besençon, France), while group B were assigned with K3 instruments (SybronEndo, West Collins, CA, USA). Hero 642 and K3 files were used with a 16:1 gear reduction hand piece powered by a torque-controlled electric motor (TCM EndoIV; Nouvag AG, Goldach, Switzerland) at a speed of 300 rpm using a crown-down technique.

The sequences of instrumentation are summarized in Table I. The sequence of Hero 642 was modified by omitting the .02T. First, size 30 .06 and .04T files were used to enlarge the coronal third of the root canal. Next, a size 25 .06T file was used to prepare the coronal segment of the root canal, followed by a size 25 .04T file to smoothly reach the working length, with a size 30 .04T file as the master apical. The instruments were gently moved apically in a circumferential brushing motion until resistance was felt. No pecking motion or apical pressure was applied. The canal patency was examined with a size 8 K-file in order to assure no dentin debris remained. The total number of times each set of instruments was used was 15. All instrumentations were performed by the same operator that was experienced in the use of Hero 642 and K3 instruments.

### Assessment of canal preparation

In the two groups, the mesiobuccal canals were first instrumented in unsectioned teeth and the change in canal curvature was recorded. The distance from the coronal stop to the tip of the file was measured, and following calibration, the distance from the tip of the file to the radiographic apex was determined. The difference between the preoperative and postoperative measurements provided the alteration in the working length. All measurements were performed by an operator blinded to the experimental grouping.

**Figure 3:**
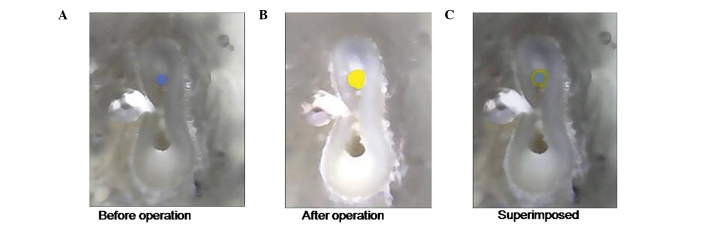
Differences between pre- and postoperative canal areas. (A) Preoperative (canal designated in blue); (B) postoperative (canal designated in yellow); (C) superimposed root cross-sections. The superimposed area was used to further calculate outcome measures.

**Figure 4:**
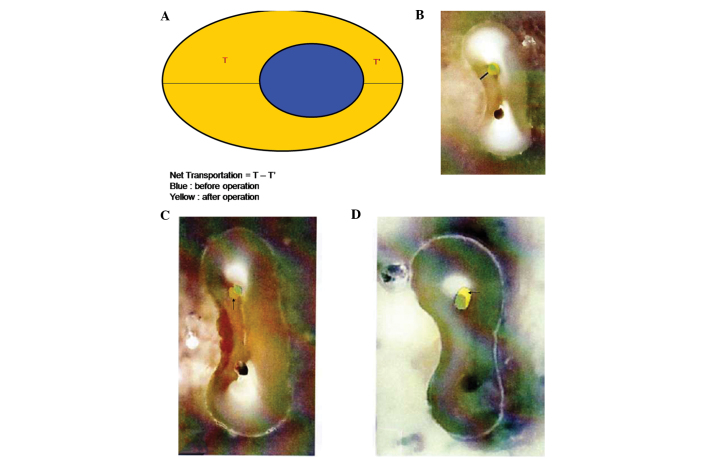
(A) Canal transportation measurements at the direction of maximum transportation (net transportation = T-T′). (B) Black line in the root cross-section indicates the remaining minimum dentin thickness following instrumentation. (C) Net transportation towards the inside of the curve (distal) at the middle level in the Hero 642 group. (D) Net transportation towards the outside of the curve (mesial) at the apical level in the Hero 642 group.

Following the completion of mesiobuccal canal preparation, the tooth blocks were sectioned at a distance of 3 and 6 mm from the apex using a low-speed diamond saw (IsoMet Low Speed Saw; Buehler, Lake Bluff, IL, USA). The section at 3 mm from the apex was defined as the apical level and the section at 6 mm from the apex was defined as the middle level (Fig. 1E). The sections were scanned and stored as jpeg images. The blocks were reassembled in the muffle system, and the mesiolingual canals were instrumented using the same technique. The sections were again digitized, and the pre- and postoperative cross-sectional canal spaces were colored and superimposed manually using Photoshop CS3 (Adobe Systems, Inc., San Jose, CA, USA; Fig. 3). In the mesiolingual preparations, the pre- and postoperative cross-sectional areas were recorded using a computer program (AutoVue SM Pro). Sections of the teeth were evaluated for canal transportation, cross-sectional area and the minimum remaining dentin thickness. Canal transportation was measured only in the direction of maximum transportation, using the method described by Bergmans *et al* (16), via the calculation of net transportation. This was defined as T-T′, where T represented the difference in the maximum radius following root canal preparation and the radius prior to root canal preparation; T′ represented the difference in the minimum radius following root canal preparation and the radius prior to root canal preparation; and T-T′ represented the deviation from the middle position of the root canal following preparation (Fig. 4A). The cross-sectional area of each section was measured and evaluated prior to and following canal preparation, with the difference indicating the amount of dentin removed at each level. The remaining dentin thickness was measured as the distance from the outer aspect of the canal to the outer aspect of the root in all directions, with the shortest distance considered the minimum remaining dentin thickness (Fig. 4B).

### Canal straightening

Instrument systems were assessed for canal straightening on patients who were undergoing observations for root canal treatment in the Department of Endodontics of the Second Affiliated Hospital of Sun Yat-sen University (Guangzhou, China). Twenty canals were instrumented with the Hero 642 instruments and 20 canals with the K3 system, which were then evaluated for the degree of straightening. The canals were mesial canals of mandibular molars or mesiobuccal canals of maxillary molars. Canal preparation was performed according to the aforementioned technique to an apical size of 30 and .04T. The pre- and postoperative radiographs were digitized, and the degree of canal straightening was calculated for the two groups. The study was approved by the Institutional Review Board of the Second Affiliated Hospital of the Sun Yat-sen University, and all the patients provided written informed consent.

### Statistical analysis

SPSS 11.0 software (SPSS, Inc., Chicago, IL, USA) was used for the statistical analysis. All data regarding canal straightening, alterations in the working length, transportation, cross-sectional area and dentin thickness were analyzed using the Mann-Whitney U test, where P<0.05 was considered to indicate a statistically significant difference. SPSS 11.0 software was used for the analysis (SPSS Inc, Chicago, IL, USA) was used for the statistical analyses.

### Systematic review

A search of Medline, Cochrane, EMBASE and Google Scholar databases was performed on June 30, 2013 using combinations of the following keywords: Hero 642, K3, NiTi, rotary nickel-titanium file and root canal preparation. Studies were included in the review if they met the following criteria: i) Compared Hero 642 and K3 files with use in human teeth; ii) prospective comparative or randomized study; and iii) written in the English language. Studies were identified using this search strategy by two independent reviewers. Where there was uncertainty regarding eligibility, a third reviewer was consulted.

The following information was extracted from the studies that met the inclusion criteria: Name of the first author, year of publication, study design, number of subjects, type of subjects, outcome measures and the results of the comparison between Hero 642 and K3.

## Results

### Mesiobuccal canal instrumentation

Mean preoperative angles of curvature were 19.7° in group A and 21.2° in group B (P=0.473; Table II). No statistically significant difference was identified between the preoperative and postoperative degrees of root canal curvature in the two groups (Table II). The mean degree of straightening was 3.4° in group A and 2.6° in group B (P=0.273; Table III). The mean change in working length was greater in group B compared with group A, however, the difference was not statistically significant (P=0.076; Table III). No file breakage occurred in the groups and all the canals remained patent.

### Mesiolingual canal instrumentation

In the two groups, the canals demonstrated a tendency towards inner transportation in the middle sections. With the Hero 642 system, 17 out of 18 canals were transported towards the inside of the curve (distal) in the middle 6 mm sections (Fig. 4C), and only one canal was transported towards the outside of the curve (mesial). With regard to the apical sections, nine out of 18 canals were transported towards the outside of the curve (mesial; Fig. 4D), eight canals were transported towards the inside of the curve (distal) and one canal remained centered.

With the K3 files, four canals out of 18 were transported towards the outside of the curve, while 14 canals were transported towards the inside of the curve at the middle level. At the apical level, nine canals out of 18 were transported towards the outside of the curve, eight canals were transported towards the inside and one canal remained centered. At the middle level, the mean transportation was 0.132 mm in group A and 0.141 mm in group B (P=0.894; Table III). In the apical sections, the difference in mean transportation between groups A and B was not statistically significant (0.086 vs. 0.088 mm, respectively; P=0.691; Table III).

Cross-sectional areas and the amount of dentin removed are shown in Table IV. The mean preoperative cross-sectional area was similar between groups A and B at the apical and middle levels. No statistically significant differences in the mean postoperative cross-sectional area were observed at the middle (P=0.121) or apical level (P=0.730). However, the difference between the mean pre- and postoperative cross-sectional areas was significant for groups A and B at the middle and apical levels. No statistically significant difference in the amount of dentin removed was observed between the two groups at the middle (P=0.804) or apical levels (P=0.654).

The difference between the mean pre- and postoperative dentin thickness was statistically significant in groups A and B at the middle level, but not significant at the apical level (P<0.05; Table V). Postoperatively, no statistically significant difference in the remaining minimum dentin thickness was observed between the groups at the middle (P=0.215) or apical level (P=0.344).

### Canal straightening

**Figure 5:**
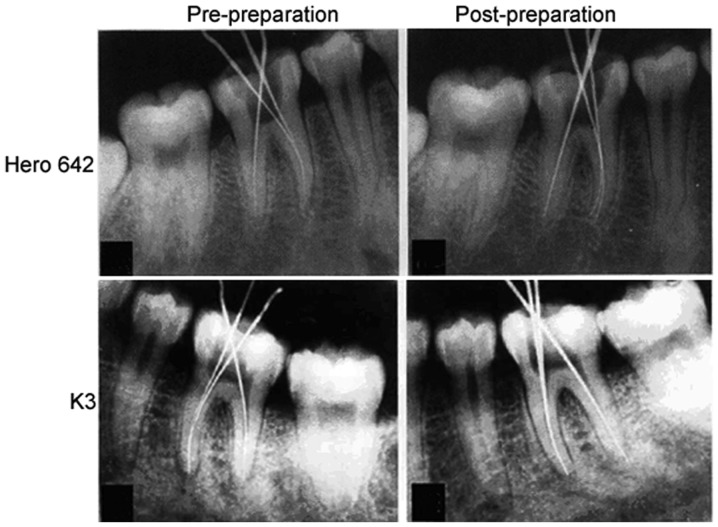
Representative radiographs of the patients treated with Hero 642 and K3 instruments.

Representative radiographies of the patients treated with Hero 642 and K3 instruments are demonstrated in Fig. 5. No statistically significant difference in the preoperative angle of curvature was observed between the canals in the two groups (P>0.05). Postoperatively, the average angle of curvature was significantly decreased in the Hero 642 (P=0.022) and K3 groups (P=0.045; Table VI). The average degrees of canal straightening in the Hero 642 and K3 groups were 4.4±2.7 and 3.4±3.5°, respectively (P=0.213).

### Systematic review

A total of 197 studies were identified using the search criteria. Following the exclusion of non-relevant studies and those that did not meet the inclusion criteria, six studies were included in the review (17–22). The characteristics of the studies are shown in Table VII. Overall, Hero 642 and K3 files performed similarly in the categories examined. Notably, Prati *et al* (22) performed scanning electron microscopy to evaluate the ultrastructural morphology of root canal walls and identified that the K3 group had marked pulpal debris in the apical third when compared with the Hero 642 group. However, De-Deus *et al* (18) did not identify a statistically significant difference in the remaining pulp tissue between the Hero 642 and K3 groups when using routine microscopic histological examinations. Guelzow *et al* (20) reported that the working time for Hero 642 files was greater compared with the K3 files, however, the time required for changing the instruments of Hero 642 files was reduced in comparison. González-Rodríguez *et al* (19) reported that the mean area of dentin removal was greater with Hero 642 files than with K3 files.

## Discussion

The present study found that Hero 642 and K3 instrument systems prepared root canals similarly with no file breakage. The root canals were prepared to an acceptable size and the original direction of the roots was preserved. The systematic review of studies comparing Hero 642 and K3 files found that the two files performed similarly in the majority of categories examined; however, differences were observed with regard to the working time, the time required for changing the instrument and the amount of dentin removed. The working lengths and canal straightening determined in the present study were similar to the results of previous studies; however, the amount of dentin removed differed from that reported by González-Rodríguez *et al* (19).

The results of the present study are in general accordance with other studies, affirming that NiTi files maintain the canal curvature (9,20,23). Jodway and Hülsmann reported a mean straightening of 0.4° for the K3 instrument (9), which is considerably less compared with the value reported in the present study, but highlights the minimal change in the curvature with NiTi files. Root canal straightening may reflect the file tip design and the natural tendency of NiTi files to straighten due to their elastic property (8). The K3 system has demonstrated advantages in preparing S-shaped root canals, possibly due to the cross-sectional design and sequence encompassing a high number of instruments (8).

Previous studies have hypothesized that Hero 642 instruments do not prepare canals to a form with sufficient taper (5). Therefore, the .02T was omitted to allow larger and more tapered files to reach the apex, and also to enable direct comparison with the K3 instruments. This modification was favorable and the Hero 642 system performed similarly to that reported in other studies (24). With the two instruments there was a tendency towards inner transportation in the middle sections. Previous studies have reported canal transportation (5,8,16,20,25); however, the clinical significance of small canal transportation has also been questioned (26).

No file breakage occurred in the present study, indicating that file breakage may be avoided if the files are carefully handled without the use of high pressure and timely irrigated. Previous studies have also reported the relative safety of these instruments during instrumentation (9,27).

Hero 642 files have been reported to remove more dentin than K3 files (19), and K3 files are associated with greater remaining dentin thickness compared with a number of other instruments (28,29). In the present study, no statistically significant difference was observed between the two instruments with regard to the amount of dentin removed. By contrast, González-Rodrguez *et al* (19) reported that Hero 642 files removed a greater mean area of dentin compared with K3 files. With respect to canal straightening, Hero 642 and K3 files were found to effectively decrease the angle of curvature, but there was no statistically significant difference in the average canal straightening.

The present study had several limitations. Firstly, the Hero 642 instrument sequence was modified from the manufacturer’s recommendations. In addition, microscopic investigation of the canal walls to demonstrate debris removal was not performed. Furthermore, the teeth studied were restricted to an ethnic southern Chinese population and the sample size was relatively small. Therefore, generalizability of the results may be limited and further studies are required to determine their clinical utility.

In conclusion, the results of the present study and the systematic review indicate that the Hero 642 and K3 rotary NiTi instrument systems were comparable with respect to their centering ability, effect on the working length, amount of dentin removed and the minimum dentin thickness remaining following preparation. Although the two systems are of different designs, their results are concordant.

## Figures and Tables

**Table I:** Instrumentation sequences for Hero 642 and K3.

Instrumentation system	Sequence of instruments	Steps
Hero 642 instruments	Size 30 .06T file	Preparing canals until encountering resistance
	Size 30 .04T file	
	Size 25 .06T file	
	Size 25 .04T file	Preparing canals until reaching working length
	Size 30 .04T file	
K3 instruments	Size 25 10T file	Opening orifice
	Size 25 .08T file	
	Size 30 .06T file	Preparing canals until encountering resistance
	Size 30 .04T file	
	Size 25 .06T file	
	Size 25 .04T file	Preparing canals until reaching working length
	Size 30 .04T file	

**Table II:** Pre- and postoperative root canal curvatures in the mesiobuccal instrumentation.

	Angle of curvature (°)		
		
Group	Preoperative	Postoperative	P-value
Hero 642 (n=20)	19.7±6.2^a^	16.2±6.5	0.091
K3 (n=20)	21.2±6.9^a^	18.7±6.1	0.239

Data are presented as the mean ± standard deviation.

a P=0.473, indicating no significant difference in the preoperative angle of curvature between the two groups.

**Table III:** Canal straightening, change in working length and canal transportation in the mesiolingual instrumentation.

Parameter	Hero 642 (n=20)	K3 (n=20)	P-value
Canal straightening (°)	3.4±2.5	2.6±1.9	0.273
Change in working length (mm)	0.151±0.106	0.223±0.138	0.076
Canal transportation (mm)			
Middle level	0.132±0.075	0.141±0.098	0.894
Apical level	0.086±0.069	0.088±0.044	0.691

Data are presented as the mean ± standard deviation.

**Table IV:** Mean pre- and postoperative cross-sectional areas and amount of dentin removed in the mesiolingual instrumentation.

Parameter	Hero 642 (n=20)	K3 (n=20)	P-value
Cross-sectional area (mm^2^)			
Middle level			
Preoperative	0.177±0.140	0.131±0.075	0.467
Postoperative	0.296±0.124^a^	0.254±0.075^a^	0.121
Apical level			
Preoperative	0.076±0.062	0.082±0.057	0.887
Postoperative	0.153±0.016^a^	0.143±0.044^a^	0.730
Dentin removed (mm^2^)			
Middle level	0.122±0.057	0.125±0.081	0.804
Apical level	0.075±0.041	0.063±0.031	0.654

Data are presented as the mean ± standard deviation.

a Indicates a statistically significant difference between the pre- and postoperative values.

**Table V:** Mean pre- and postoperative remaining minimum dentin thickness in the mesiolingual instrumentation.

Parameter	Hero 642 (n=20)	K3 (n=20)	P-value
Middle level			
Preoperative	0.997±0.202	0.954±0.357	0.235
Postoperative	0.821±0.255^a^	0.737±0.383^a^	0.215
Apical level			
Preoperative	0.845±0.225	0.751±0.167	0.255
Postoperative	0.777±0.221^a^	0.685±0.177^a^	0.344

Data are presented as the mean ± standard deviation.

a Indicates a statistically significant difference between the pre- and postoperative values.

**Table VI:** Angles of curvature prior to and following instrumentation.

Group	Preoperative (°)	Postoperative (°)	P-value
Hero 642 (n=20)	20.5±6.3	16.1±6.2	0.022
K3 (n=20)	22.8±6.7	19.3±6.5	0.045

**Table VII:** Characteristics of the selected studies.

First author (year)	Study design	Teeth	Teeth (n)	Instruments compared	Outcome measures/results of the comparison between Hero 642 and K3
De-Deus G (2009) (16)	*ex vivo*	Mandibular molars	67	Hero 642 K3 ProTaper	Remaining pulp tissue: Hero 642=K3
Mohammadi Z (2007) (19)	*ex vivo*	Maxillary central incisor	110	Hero 642 K3 Flex Master Profile GT RoCe Control	Bacterial colonies: Hero 642=K3
de Carvalho Maciel AC (2006) (15)	*ex vivo*	Single-rooted teeth	100	Hero 642 K3 ProFile ProTaper Manual	Amount of filling debris remaining on the canal walls: Hero 642=K3
Guelzow A (2005) (18)	*ex vivo*	Mandibular molars	147	Hero 642 K3 ProTaper RaCe Flex Master System GT Manual	Working time: Hero 642>K3 Time for changing instruments: Hero 642<K3 Working length: Hero 642=K3 Straightening of curved root canals: Hero 642=K3 Postoperative root canal diameter: Hero 642=K3 Procedural incidents: Hero 642=K3
Prati C (2004) (20)	*in vitro*	Maxillary incisors	48	Hero 642 K3 RaCe K-file Manual	Pulpal debris: Hero 642<K3 Presence of smear layer: Hero 642=K3 Inorganic debris: Hero 642=K3 Surface profile: Hero 642=K3
González-Rodríguez MP (2004) (17)	*in vitro*	Mandibular molars	34	Hero 642 K3 Profile	Mean area of dentin removed: Hero 642>K3 Mean initial area of the canal: Hero 642=K3
Present study	*ex vivo*	Mandibular molars	40	Hero 642 K3	Canal straightening: Hero 642=K3 Change in working length: Hero 642=K3 Canal transportation: Hero 642=K3 Amount of dentin removed: Hero 642=K3 Remaining minimum dentin thickness: Hero 642=K3
